# Letter on “Left ventricular systolic function evaluated by strain echocardiography and relationship with mortality in patients with severe sepsis or septic shock: a systematic review and meta-analysis”

**DOI:** 10.1186/s13054-019-2312-1

**Published:** 2019-02-08

**Authors:** Venu M. Velagapudi, Dennis A. Tighe

**Affiliations:** 1 Division of Pulmonary and Critical Care Medicine, Yale University School of Medicine, New Haven, CT USA; 2 Division of Cardiovascular Medicine, University of Massachusetts Medical School, Worcester, MA USA

We followed with interest the study by Sanfilippo et al. [1], a meta-analysis of pooled observational studies of severe sepsis and/or septic shock. The authors included 794 patients stratified by survivors/non-survivor and showed that global longitudinal strain (GLS) measurements were strongly associated with survival (standard mean difference (SMD) − 0.26; 95% confidence interval (CI) − 0.47, − 0.04; *p* = 0.02).

We bring to your attention the significant inherent variability of GLS measurements in the context of proprietary differences in image acquisition platforms and inter-vendor variability in speckle tracking algorithms.

As the pooled data was comprised of four different vendor platforms, interpretation of pooled results should be done with abundant caution. It is notable that when the authors [1] attempted sensitivity analysis grouping studies by vendors/software, such analysis was deemed not feasible.

We particularly emphasize that GLS standard mean difference (SMD) − 0.26 and 95% confidence interval (CI) − 0.47 and − 0.04 between survivors and non-survivors cited in the study [1] should be interpreted with caution in the context of the existing literature: (a) Absolute difference between vendors for average GLS from the three apical views was up to 3.7% strain units [2] and (b) inter-vendor 2D speckle tracking software variability limits of agreement range ± 3 to ± 4.5% in measuring GLS [3].

The pooled standard mean difference was less than known inter-vendor variability.

Furthermore, longitudinal deformation is highest in the endocardium and lowest in the epicardium. Currently, there is insufficient evidence to decide if the endocardial, mid-wall, or full-wall strain is the best choice for clinical use [4].

Although the methodology of meta-analysis is robust, the inherent variations in vendor protocols to measure strain, layer specific nature of strain measurements, patient characteristics, and known technical limitations in acquiring strain measurements limit the generalizability of results of such pooled data.

Another systematic review of GLS in severe sepsis/septic shock [5] which analyzed a total of 455 patients did not combine the data by using meta-analysis methods citing significant methodological and statistical differences between the studies.

Until GLS measurements undergo further standardization in the future as being proposed by European Association of Cardiovascular Imaging (EACVI)-American Society of Echocardiography (ASE) strain standardization task force, this limitation continues to apply to currently available pooled data.
