# Supplementary material for: Letter on “Left ventricular systolic function evaluated by strain echocardiography and relationship with mortality in patients with severe sepsis or septic shock: a systematic review and meta-analysis”
Source: Crit Care. 2019 Feb 8;23:38. doi: 10.1186/s13054-019-2312-1 (PMC6368704; doi:10.1186/s13054-019-2312-1)
Supplement: Related texts

# Reply: Authors’ response

**Authors:** Filippo Sanfilippo, Carlos Corredor, Nick Fletcher, Luigi Tritapepe, Ferdinando L. Lorini, Antonio Arcadipane, Antoine Vieillard-Baron, Maurizio Cecconi

We thank Dr. Velagapudi and Tighe for their interest in our paper and for highlighting one of its limitations [1]. Differences between inter-vendor software for evaluation of global longitudinal strain (GLS) were discussed in our study as one of the issues preventing to draw firm conclusions on our results. However, despite such variabilities, we believe that our results are still valid since the goal of studies included into our meta-analysis was not to give any cutoff value of strain that could differentiate between survivors and non-survivors, but rather to study the link between strain as a continuous value and prognosis.

However, in truth, there are other limitations in this and other meta-analyses in the field of echocardiography [1, 6–8]. Among these, we believe that the most important one remains the lack of correction for confounding factors because these adjustments were never (or rarely) reported in the original studies included. Therefore, in common with most meta-analyses, our meta-analysis only suggests “associations” without the possibility to draw firm conclusions about “causative” mechanisms between the echocardiographic findings and outcomes. Nonetheless, these meta-analyses have also several advantages; the most important we believe are (1) suggesting directions for further echocardiography research through detection of hypothesis-generating associations and (2) highlighting the need for more structured reporting in the setting of critical care echocardiography research.

On a separate note, the correspondents also discuss in their letter the relevance of a small standardized mean difference (SMD) − 0.26 (95% confidence interval − 0.47, − 0.04) in the context of the already mentioned inter-vendor differences. It should be noted that, even if SMD does not correct for the variability between softwares, the Cochrane Handbook states that SMD is used in a meta-analysis when studies assess the same outcome but measure it in a variety of ways (i.e., using different scales). In these circumstances, SMD is necessary to standardize the results of the studies to a uniform scale before they can be combined. The SMD expresses the size of the intervention effect in each study relative to the variability observed in that study [9]. Therefore, the statistical approach we used was the most appropriate to account for the variability of GLS. Furthermore, there are grounds to hypothesize that the use of SMD may have reduced the impact of inter-vendor differences.

More research is needed in critical care echocardiography; meta-analyses of non-randomized studies should not be taken at face value but as a driving force for further well-designed and focused research.
